# Clinical value of miR-551b-5p in children with primary nephrotic syndrome and its regulatory role in disease progression

**DOI:** 10.1186/s41065-026-00644-3

**Published:** 2026-02-09

**Authors:** Yuehong Yang, Lijun Zhao, Fang Wu, Caihong Xue, Xiaoyun Qu

**Affiliations:** Department of Nephrology, Shanxi Children’s Hospital, No. 13, Xinmin North Street, Xinghualing District, Taiyuan, 030013 China

**Keywords:** MiR-551b-5p, CD2AP, Primary nephrotic syndrome, Biomarker, Podocyte

## Abstract

**Background:**

Primary nephrotic syndrome (PNS) is a common glomerular disorder in children. Despite significant advances in glucocorticoid therapy, considerable challenges remain in the early diagnosis and prognostic management of PNS. Elevated miR-551b-5p expression has been detected in the PNS. This study aims to further explore the role of miR-551b-5p in the onset and progression of PNS and its potential mechanisms.

**Methods:**

A total of 107 patients with primary nephrotic syndrome (PNS) and 99 healthy volunteers (HV) were included in this study. And the PNS group was further divided into subgroups with favorable (*n* = 76) and poor (*n* = 31) prognosis. The expression of miR-551b-5p in the PNS and the poor prognosis group was quantified using qRT-PCR. The predictive capability of miR-551b-5p for the occurrence and poor prognosis of PNS was evaluated. The effects of miR-551b-5p knockdown on podocyte growth and inflammatory injury were examined. The interaction between miR-551b-5p and CD2AP was verified via database and luciferase assay.

**Results:**

miR-551b-5p is obviously elevated in PNS and the poor prognosis group. miR-551b-5p exhibits strong diagnostic capability for both the onset and poor prognosis of pediatric PNS. High miR-551b-5p expression is an independent risk factor for poor prognosis in pediatric PNS patients. miR-551b-5p downregulation potently promotes podocyte proliferation and reduces apoptosis, and improves intracellular inflammatory responses and oxidative stress levels. Furthermore, CD2AP is a direct target of miR-551b-5p, and this regulatory axis synergistically contributes to the pathogenesis and progression of PNS.

**Conclusion:**

miR-551b-5p is a potential biomarker for predicting the occurrence and poor prognosis of PNS. miR-551b-5p promotes the onset and progression of PNS by targeting CD2AP.

**Supplementary Information:**

The online version contains supplementary material available at 10.1186/s41065-026-00644-3.

## Introduction

Primary nephrotic syndrome (PNS) is a highly prevalent glomerular disorder. It is responsible for about 90% of all pediatric nephrotic syndrome (NS) cases [1]. It is characterized by massive proteinuria, hypoalbuminemia, edema, and hyperlipidemia resulting from increased permeability of the glomerular filtration membrane to plasma albumin (Alb) [2, 3]. Glucocorticoids are the primary treatment for PNS. Despite a generally favorable response in most children, a significant proportion experience complications such as recurrent relapses, glucocorticoid dependence, or even resistance. And these patients are also at high risk of developing end-stage renal disease [4]. Therefore, identifying biomarkers associated with PNS is crucial for early diagnosis and prognostic management.

MicroRNAs (miRNAs) as biomarkers have shown great potential in the pathogenesis and prognosis of NS. For instance, Wang et al. reported that elevated urinary exosomal miR-193a correlates with increased incidence of primary focal segmental glomerulosclerosis in childhood NS and heightened risk of poor prognosis [5]. Xu et al. indicated that miR-151-3p influences the onset and progression of NS by targeting GLCCI1 [6]. Previous studies have mentioned that the upregulation of miR-551b-5p may be linked to poorer prognosis in childhood NS [7]. However, its role in PNS has yet to be fully proved.

CD2-associated protein (CD2AP), encoded by the CD2AP gene, is a key component of the glomerular filtration barrier and plays a crucial role in the structure and function of podocytes [8]. Dysregulation of CD2AP expression is associated with the pathogenesis of multiple diseases. For instance, Yan et al. revealed that CD2AP deficiency activates p38 MAPK, thereby exacerbating Alzheimer’s disease pathology [9]. According to Zhang et al., CD2AP promotes glioblastoma progression by activating TRIM5-dependent NF-kB signaling [10]. CD2AP gene mutations are linked to focal segmental glomerulosclerosis and serve as a potential prognostic biomarker in renal clear cell carcinoma [11, 12]. Bioinformatic analysis identified CD2AP as a target of miR-551b-5p; their collaborative role in the progression of PNS warrants further exploration.

In this study, the predictive capability of serum miR-551b-5p for the onset and poor prognosis of PNS was evaluated. The influence of miR-551b-5p on podocyte proliferation, apoptotic activity, and inflammatory injury via cell experiments, aiming to elucidate its contribution to the pathogenesis of PNS. Furthermore, the regulatory relationship between miR-551b-5p and its downstream target CD2AP was confirmed using bioinformatics prediction and dual-luciferase reporter assays. Their collaborative role in the development of PNS was further explored to provide novel targets for PNS diagnosis and prognostic evaluation.

## Materials and methods

### Clinical samples

Peripheral venous blood was collected from 107 PNS patients and 99 healthy volunteers (HV) at Shanxi Children’s Hospital from April 2019 to May 2021. Blood samples were collected from pediatric patients with PNS on the morning of the day following admission (after diagnosis and before initiating any immunosuppressive therapy). This study protocol was reviewed and approved by the Ethics Committee of Shanxi Children’s Hospital and Shanxi Children’s Hospital. Informed consent was obtained in writing from the legal guardian of each participant. The inclusion criteria comprised: (a) The patient presented with massive proteinuria, hypoalbuminemia, edema, and hyperlipidemia. And renal biopsy findings met the diagnostic criteria for PNS [13]; (b) The patient does not have secondary nephrotic syndrome or any other type of nephropathy. (c) The patient was newly diagnosed with PNS, had not taken any medications such as immunosuppressants, lipid-lowering agents, or hormones before the onset of symptoms. (d) Age ≤ 12 years old. (e) Undergoing glucocorticoid therapy, with no contraindications to treatment (recurrent relapses, glucocorticoid dependence, or even resistance). And exclusion criteria: (a) Familial dyslipidemia; (b) Patients with concomitant autoimmune diseases or malignancies; (c) Patients with concomitant cardiovascular or cerebrovascular diseases, or severe infections; (d) Patients with impaired cardiac, hepatic, or pulmonary function.

### Follow-up and prognostic assessment

The prognosis of PNS children was followed up for 3 years after treatment by telephone or outpatient review. Based on follow-up criteria and PNS outcome standards, 107 PNS patients were categorized into a favorable prognosis subgroup (partial remission, complete remission, and clinical cure) and a poor prognosis subgroup (no remission). The basis for prognosis assessment is as follows [13]: (1) Partial remission. Morning urine protein reduction > 50%, serum Alb > 25 g/L, renal function essentially stable. (2) Complete remission. Normal blood biochemistry and urinalysis. (3) Clinical cure. Long-term stable renal function with no recurrence for over 3 years after discontinuing medication. (4) No remission. Morning urine protein reduction < 50%, persistent deterioration of renal function.

### Cell culture and transfection

The MPC-5 cell suspension was sourced from EK Bioscience (Shanghai, China, Cat# CC-Y1716) and cultured in DMEM medium (Gibco, Cat# 11966025) supplemented with 10% FBS (Gibco, Cat# 10091148), 100 U/mL penicillin, and 100 µg/mL streptomycin at 37 °C under 5% CO₂. After 7 days, inflammatory injury was induced by treating the cells with 1 µg/mL lipopolysaccharide (LPS; Sigma-Aldrich, USA, Cat# L2630) for 12 h. miR-551b-5p mimics (GAAAUCAAGCGUGGGUGAGACC), miR-551b-5p inhibitors (GGUCUCACCCACGCUUGAUUUC), CD2AP siRNA, and corresponding negative controls (NC, 5’-CAGUACUUUUGUGUAGUACAA-3’) were obtained from RiboBio (Guangzhou, China) and transfected into podocytes using Lipofectamine 2000 (Invitrogen, Thermo Fisher Scientific, Cat# 11668019).

### Extraction of total RNA and qRT-PCR

After allowing the blood to stand at room temperature for 30 min, it was centrifuged at 3000 rpm for 10 min at 4 °C using a refrigerated ultracentrifuge (Beckman Coulter Optima XPN-100 ultracentrifuge, Beckman Coulter). The resulting serum was then aliquoted and stored at −80°C for subsequent use. All samples were thawed only once. RNA quality was assessed using the Agilent 2100 Bioanalyzer with the RNA Nano 6000 chip, confirming normal profiles. TRIzol reagent (Solarbio, Beijing, China, Cat# R1100) was applied for extracting total RNA. Free hemoglobin absorbance in samples was measured at 414 nm using a NanoDrop 1000 spectrophotometer (Thermo Scientific, Cat# ND2000). A threshold of > 0.2 arbitrary units at A414 was set to indicate hemolysis{Kirschner, 2011 #1129}. All hemolyzed samples were excluded from final analysis to ensure miRNA profiles remained unaffected by erythrocyte-derived miRNAs. cDNA was synthesized and qPCR detection for miR-551b-5p was conducted via the TaqMan reverse transcription kit (ThermoFisher Scientific, Cat# 4366596) and TaqMan miRNA Assays (Thermo Fisher Scientific, Cat# 4427975). Reverse transcription and the mRNA level of CD2AP using M-MLV Reverse Transcriptase (Thermo Fisher Scientific, Invitrogen, Cat# 28025013) and 2×SYBR Green PCR Mastermix (Solarbio, Beijing, China, Cat# SY102). The reverse transcription conditions were 25 °C for 30 min, 42 °C for 30 min, and subsequently 85 °C for 5 min. And the PCR amplification was performed using the following conditions: 95 °C for 30 s, followed by 39 cycles at 95 °C for 5 s, 60 °C for 30 s, and 72 °C for 15s. miR-551b-5p (forward primer 5’-GTCGTATCCAGTGCAGGGTCCGAGGTATTCGCACTGGATACGACGGTCTC-3’ and reverse primer 5’-GCAGGGTCCGAGGTATTC-3’); CD2AP (forward primer 5’-CAAGATGCCTGGAAGACGA-3’ and reverse primer 5’-GCACTTGAAGGTGTTGAAAGAG-3’). Next, U6 (forward 5’-CTCGCTTCGGCAGCACA-3’ and reverse 5’-AACGCTTCACGAATTTGCGT-3’) and GAPDH (forward 5’-ACTGGCATGGCCTTCCGT-3’ and reverse 5’-CCACCCTGTTGCTGTAGCC-3’) were used as controls for miR-551b-5p and CD2AP. All amplifications in this study exhibited efficiency ranging from 90% to 110%, with the linear regression correlation coefficient of the standard curve exceeding 0.990. And their expressions were quantified through the 2^−∆∆ct^ method. All experiments were independently replicated at least three times to ensure reproducibility of results. The intra-batch CV averaged 0.8%. The inter-batch CV for quality control samples was 7.2%.

### Dual-luciferase reporter assay

The wild-type (WT) or mutated (MUT) sequences of miR-551b-5p containing the binding sites of CD2AP were designed by Gene Pharma (China) and inserted into the pGL3 luciferase vector (Promega, Madison, WI, USA, Cat# E1751). MPC-5 cells were co-transfected with miR-551b-5p-WT/MUT and CD2AP inhibitor/CD2AP mimic/mimic-NC/inhibitor-NC by lipofectamine 2000 (Invitrogen, Thermo Fisher Scientific, Cat# 11668019). Cell lysates were collected after 48 h of transfection, and the relative luciferase activity was quantified by normalizing firefly luminescence to Renilla luminescence with the dual-luciferase reporting kit (Promega, Shanghai, China, Cat# E1910). Each independent experiment included three biological replicates.

### CCK8 assay

The transfected cells (1 × 10⁴) were trypsinized, seeded into 96-well plates, and incubated for 24, 48, or 72 h. Subsequently, 10 µL of CCK-8 reagent was added to each well, followed by incubation at 37 °C for 2 h. Cell viability was measured using a CCK-8 assay kit (Beyotime, Shanghai, China, Cat# C0042), and the absorbance at 450 nm was detected with a microplate reader (BMG LABTECH, Offenburg, Germany).

### Flow cytometry

The Annexin V-FITC Apoptosis Detection Kit (BD Biosciences) was used to evaluate apoptosis in MPC-5 cells. After 48 h of transfection, the cells were trypsinized, harvested, and stained with Annexin V-FITC and propidium iodide (PI) for 15 min under light-protected conditions. Subsequently, the cells were centrifuged, washed twice with PBS, and analyzed by FACScan flow cytometry (BD Biosciences, USA) within 1 h.

### Detection of inflammation and oxidative stress index

LPS-stimulated MPC-5 cells were centrifuged at approximately 10⁶ cells per tube, resuspended in 450 µL PBS, homogenized, and then centrifuged at 10,000 rpm for 10 min at 4 °C. The collected supernatant was stored on ice. The concentrations of interleukin-6 (IL-6) and tumour necrosis factor-α (TNF-α) were quantified using enzyme-linked immunosorbent assay (ELISA) kits from the Nanjing Institute of Bioengineering, China. Meanwhile, the levels of malondialdehyde (MDA) and the activity of superoxide dismutase (SOD) were measured with ELISA kits supplied by Wuhan Saipei Biotechnology Co., Ltd., China. Following cessation of the color development, a microplate reader (BMG LABTECH, Offenburg, Germany) was employed to assess the optical density at 450 nm. All measurements were performed in triplicate for each well.

### Statistical analysis

All data were processed using SPSS version 23 (New York, USA) and GraphPad Prism 10.0 (California, USA), with continuous variables reported in terms of mean ± standard deviation. Intergroup clinical and pathological characteristics were analyzed using chi-square and t-tests. The association between miR-551b-5p and CD2AP expression was assessed using Pearson’s correlation coefficient. ROC analysis revealed the diagnostic value of miR-551b-5p, and multivariate logistic regression assessed its predictive value for poor prognosis in PNS. Two groups that satisfied the normality test are compared using the t-test. Univariate ANOVA was employed for multiple comparisons between groups that passed the homogeneity test for variances, while two-way ANOVA compared cell proliferation capacity. Bonferroni’s correction for multiple comparisons.

## Results

### The relative expression of miR-551b-5p and its diagnostic value

**Fig. 1:**
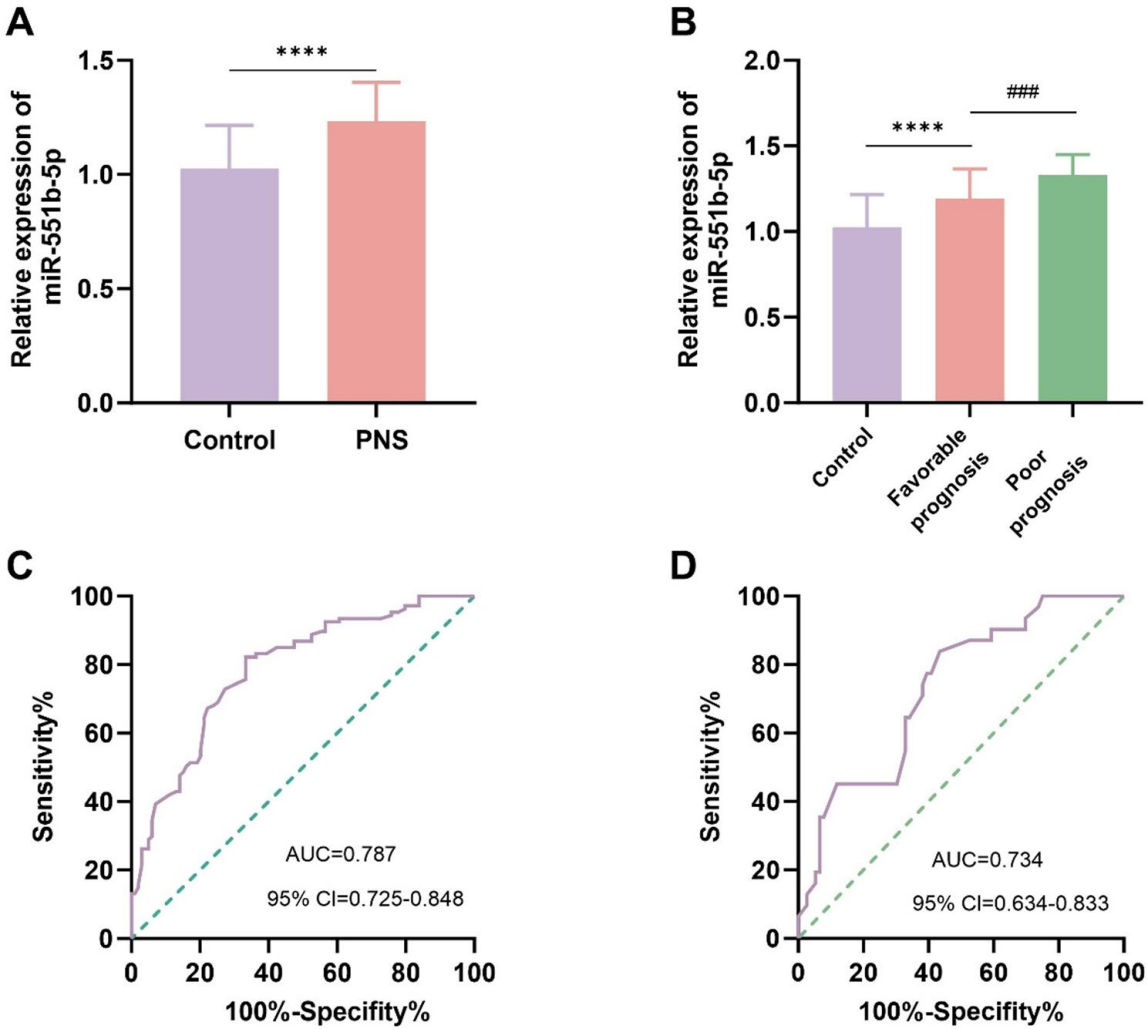
The relative expression levels of miR-551b-5p and its diagnostic value**. A** Expression of miR-551b-5p in 107 children with PNS and 99 healthy volunteers. **B** Expression of miR-551b-5p in children with PNS in the favorable and poor prognosis subgroups. **C** Serum miR-551b-5p can effectively differentiate PNS patients from healthy individuals with high accuracy, with an AUC of 0.787. **D** ROC curve for predicting poor prognosis in children with PNS based on serum miR-551b-5p levels, with an AUC of 0.734. (****P < 0.0001, ^###^*P* < 0.001)

A pronounced elevation in miR-551b-5p was discovered in PNS compared to HV (Fig. 1A). The 107 children with PNS were further divided into groups with favorable and poor prognoses. Compared with the group with favorable prognosis, the expression of miR-551b-5p was markedly upregulated in the group with poor prognosis (Fig. 1B). Serum miR-551b-5p can distinguish PNS patients from healthy individuals with high accuracy (AUC = 0.787, 95% CI: 0.725–0.848), the sensitivity and specificity were 82.24% and 66.67%, respectively (Fig. 1C). Moreover, serum miR-551b-5p also showed significant predictive value for adverse outcomes in pediatric PNS patients (AUC = 0.734, 95% CI: 0.634–0.833), sensitivity was 83.87%, specificity was 56.58% (Fig. 1D).

### Comparison of clinical characteristics between HV and patients with PNS

Clinical data for HV and PNS groups are presented in Table 1. There were no significant differences between the HV and the PNS group in terms of gender, age, BMI, Disease duration and Pathological Type (*P* > 0.05). However, compared with the HV, the PNS group exhibited higher levels of total cholesterol (TC), triglycerides (TG), blood urea nitrogen (BUN), serum creatinine (SCr), and 24-h urinary protein, as well as lower levels of Alb.

**Table 1:** Comparison of clinical characteristics between health volunteers and patients with PNS

Characteristics	HV(*n* = 99)	PNS(*n* = 107)	t(X^2^)	*P*
Gender Male Female			(0.40)	0.758
	57	71		
	42	36		
Age (years)	8.26 ± 2.41(8)	8.08 ± 2.48(8)	0.53	0.601
BMI (kg/m^2^)	21.16 ± 1.90	21.23 ± 1.87	0.27	0.789
Disease duration (d)	---	5.06 ± 1.35	---	---
Pathological Type MCD FSGS MsPGN Others				
	---	30	---	---
	---	17	---	---
	---	55	---	---
	---	5	---	---
TC (mmol/L)	4.35 ± 1.12	5.32 ± 0.95	6.76	<0.001
TG (mmol/L)	1.14 ± 0.40	2.32 ± 1.40	8.08	<0.001
Alb (g/L)	40.26 ± 7.99	21.84 ± 1.94	23.00	<0.001
BUN (mmol/L)	4.50 ± 0.89	7.27 ± 1.11	19.76	<0.001
SCr (µmol/༬)	66.48 ± 13.66	100.40 ± 15.42	16.68	<0.001
24-h urine protein (g)	---	3.86 ± 0.64	---	---

*BMI*, body mass index, *TC* total cholesterol, *TG* triglyceride, *BUN* blood urea nitrogen, *SCr* serum creatinine, *Alb* albumin, *MCD* Minimal Change Disease, *FSGS* Focal Segmental Glomerulosclerosis, *MsPGN* Mesangial Proliferative Glomerulonephritis

### Correlation of serum miR-551b-5p expression with serum, blood lipids, and renal function markers

Pearson correlation analysis revealed that serum miR-551b-5p levels were positively correlated with blood lipid markers (TC and TG) and renal function markers (BUN, SCr, or 24-h urinary protein), and significantly negatively correlated with serum Alb levels (*P*<0.001, Table 2).

**Table 2:** Correlation between serum miR-551b-5p levels and serum, lipid, and renal function markers

Indicators	miR-551b-5p	
	*R*	*P*
TC	0.585	<0.001
TG	0.587	<0.001
Alb	−0.604	<0.001
BUN	0.590	<0.001
SCr	0.577	<0.001
24- hour urine protein	0.625	<0.001

*TC* total cholesterol, *TG* triglyceride, *Alb* albumin,*BUN* blood urea nitrogen, *SCr* serum creatinine

### Comparison of clinical characteristics in children with PNS of different prognoses

Among 107 children with PNS, 31 cases showed no remission; 26 achieved partial remission, 29 attained complete remission, and 21 were clinical cures. Consequently, the 107 cases were categorized into a favorable prognosis group (76 cases) and a poor prognosis group (31 cases). As shown in Table 3, in the poor prognosis subgroup, BUN and 24-h urine protein were all higher than in the favorable prognosis subgroup, while serum Alb levels were lower.

**Table 3:** Comparison of clinical features of PNS children with different prognosis

Characteristics	PNS group (*N* = 107)		t(X^2^)	*P*
	good prognosis (*n* = 76)	poor prognosis (*n* = 31)		
Gender Male Female			(0.012)	0.915
	40	17		
	36	16		
Age	8.13 ± 2.51(8)	7.97 ± 2.44(9)	0.309	0.758
BMI (kg/m^2^)	21.12 ± 1.91	21.51 ± 1.76	0.996	0.321
Disease duration	5.06 ± 1.27	5.07 ± 1.54	0.019	0.985
Pathological Type MCD FSGS MsPGN Others			(6.012)	0.111
	20	4		
	12	11		
	40	15		
	4	1		
TC(mmol/L)	5.25 ± 0.87	5.50 ± 1.12	1.228	0.222
TG(mmol/L)	2.26 ± 1.40	2.46 ± 1.42	0.648	0.519
Alb(g/L)	28.88 ± 6.77	25.39 ± 5.55	2.539	0.013*
BUN(mmol/L)	7.11 ± 1.23	7.69 ± 0.57	2.528	0.013*
SCr (µmol/༬)	99.90 ± 15.23	101.80 ± 16.06	0.574	0.567
24-h urine protein(g)	3.17 ± 0.56	3.70 ± 0.68	4.142	0.000*
miR-551b-5p	1.19 ± 0.17	1.33 ± 0.12	4.033	0.000*

*BMI* body mass index, *MCD* Minimal Change Disease, *FSGS* Focal Segmental Glomerulosclerosis, *MsPGN* Mesangial Proliferative Glomerulonephritis, *TC* total cholesterol, *TG* triglyceride, *Alb* albumin, *BUN* blood urea nitrogen, *SCr* serum creatinine

*P<0.05

### Factors influencing poor prognosis in children with PNS

Multivariate logistic regression analysis was performed using the prognosis of children with PNS as the dependent variable (favorable = 0, poor = 1), with variables showing significant differences in Table 3 as independent variables. Results indicated that elevated miR-551b-5p levels were independent risk factors for poor prognosis in pediatric PNS patients (Table 4).

**Table 4:** Multivariate logistic regression analysis of factors affecting poor prognosis in PNS patients

Parameters	*P*	OR	95% CI
Alb (g/L)	0.354	0.629	0.230–1.670
BUN (mmol/L)	0.313	1.676	0.622–4.729
24-h urine protein (g)	0.253	1.789	0.662–4.956
miR-551b-5p	0.031*	3.201	1.146–9.672

*Alb* albumin, *BUN* blood urea nitrogen

### Effects of miR-551b-5p knockdown on podocyte growth and inflammatory injury

**Fig. 2:**
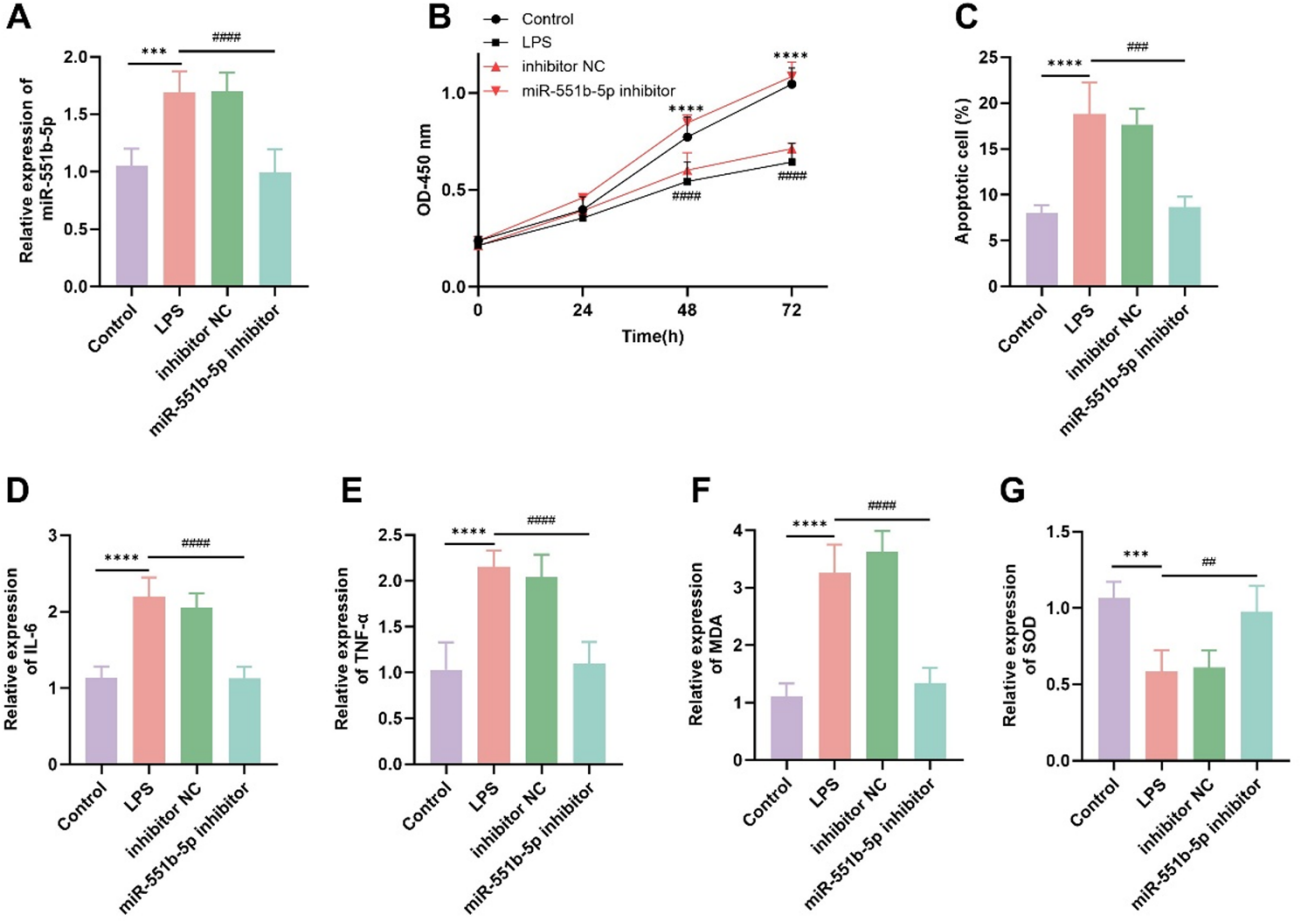
Effects of miR-551b-5p on LPS-induced proliferation and apoptosis of MPC-5 cells, as well as the expression of inflammatory mediators and oxidative stress markers.** A** miR-551b-5p expression is significantly upregulated in LPS-induced MPC-5 cells, while miR-551b-5p knockdown partially reversed the effect. **B** The suppressive effect of LPS-induced on MPC-5 cells’ proliferation was rescued by miR-551b-5p knockdown. **C** The miR-551b-5p knockdown could attenuate the increased apoptosis of MPC-5 cells caused by LPS-induced. **D **and** E** The relative expression levels of inflammatory mediators (IL-6 and TNF-α) significantly increased under LPS induction, whereas miR-551b-5p knockdown significantly restored their levels. **F **and** G** MDA levels were significantly elevated while SOD activity markedly decreased following LPS stimulation, whereas miR-551b-5p knockdown significantly restored the expression of these oxidative stress markers. (***P < 0.001, ****P < 0.0001 vs. control; ^##^*P* < 0.01, ^###^*P* < 0.001, ^####^*P* < 0.0001 vs. inhibitor-NC)

LPS stimulated MPC-5 cells to simulate an inflammatory injury model. miR-551b-5p expression is significantly upregulated in LPS-induced MPC-5 cells, while miR-551b-5p knockdown partially reversed the effect (Fig. 2A). The results of the CCK-8 assay and flow cytometry showed that LPS stimulation significantly inhibited MPC-5 cells’ proliferation and induced apoptosis, while knockdown of miR-551b-5p significantly restored cell proliferation and reduced apoptosis (Fig. 2B, C). Additionally, ELISA results revealed that the relative expression of inflammatory mediators (IL-6 and TNF-α) significantly increased under LPS induction, whereas miR-551b-5p knockdown significantly restored their levels (Fig. 2D, E). Following LPS stimulation, MDA levels significantly increased while SOD activity markedly decreased. However, miR-551b-5p knockdown significantly restored the expression of these oxidative stress markers (Fig. 2F, G).

### Interaction of miR-551b-5p with CD2AP

**Fig. 3:**
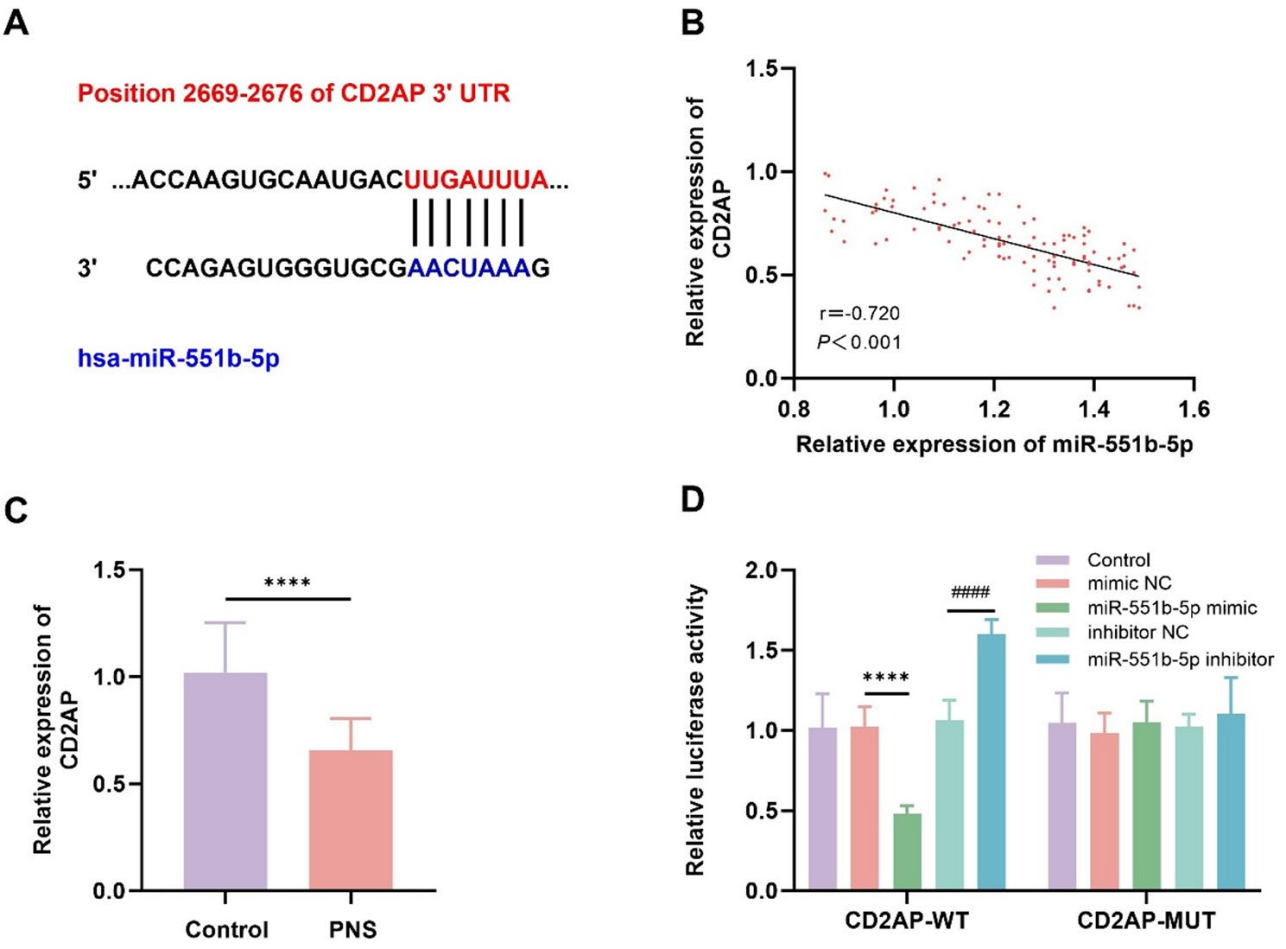
Interaction of miR-551b-5p with CD2AP.** A** CD2AP was predicted as a miR-551b-5p target via the TargetScan database. **B** miR-551b-5p expression is negatively related to CD2AP expression (*r* = − 0.720, *P* < 0.001). **C** Differential expression of CD2AP in children with PNS compared to healthy volunteers. **D** The dual-luciferase assay validated the interaction of CD2AP with miR-551b-5p. (****P < 0.0001 vs. control and mimic NC; ^####^*P* < 0.0001 vs. inhibitor NC)

miR-551b-5p could directly bind to the 3’UTR of CD2AP via the TargetScan database (https://www.targetscan.org/vert_72/) (Fig. 3A). miR-551b-5p expression is negatively related to CD2AP expression (*r* = − 0.720, *P* < 0.001, Fig. 3B). CD2AP expression was substantially reduced in PNS compared to HV (Fig. 3C). The miR-551b-5p mimic markedly suppresses CD2AP luciferase activity, while the miR-551b-5p inhibitor leads to a significant increase. However, following a mutation in CD2AP, the luciferase activity was not affected (Fig. 3D).

### miR-551b-5p and CD2AP jointly influence podocyte function and inflammatory injury

**Fig. 4:**
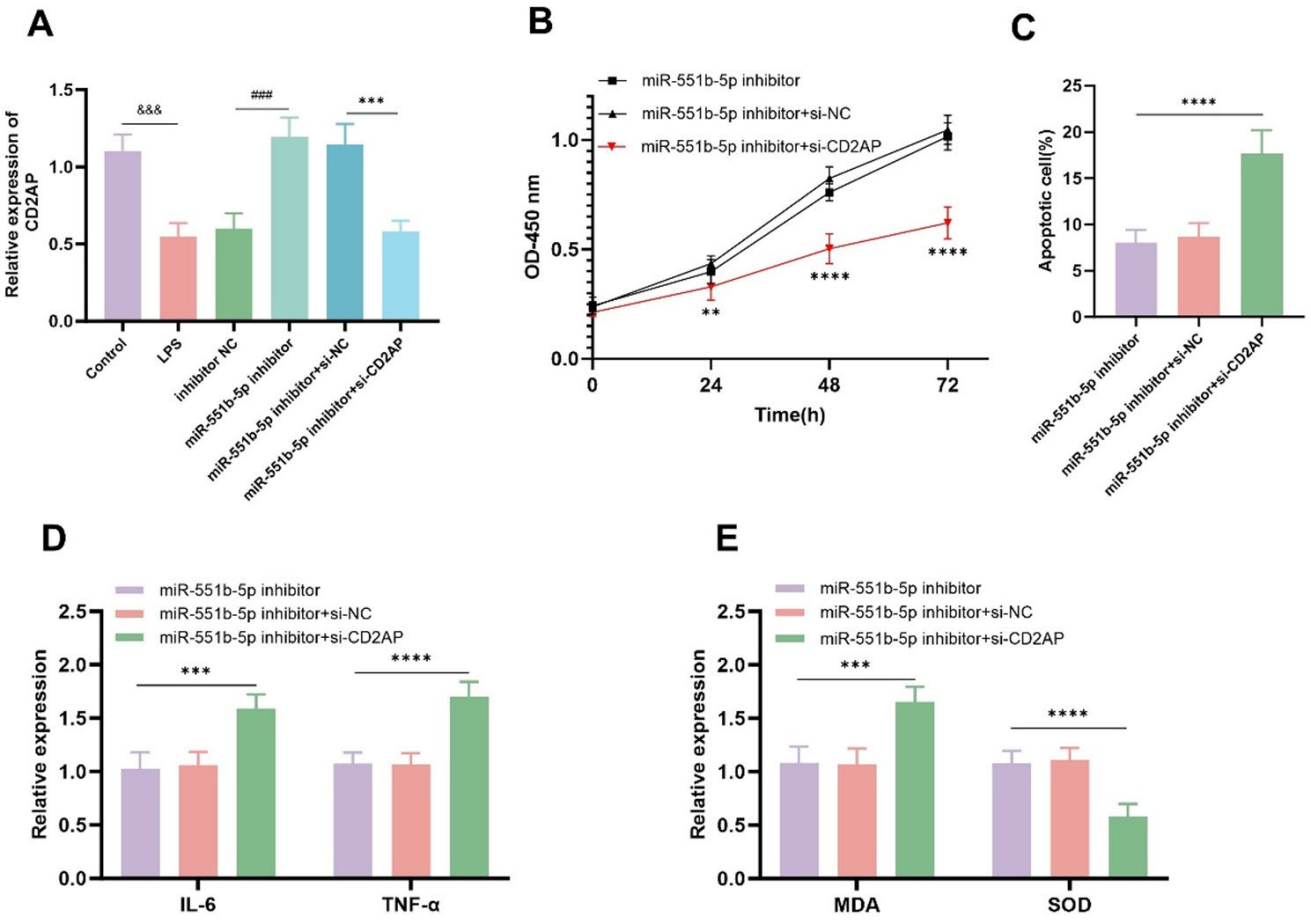
CD2AP reversed the effects of miR-551b-5p knockdown on MPC-5 cells that were subjected to LPS-induced inflammation damage.** A** The expression of CD2AP was significantly reduced upon LPS stimulation. However, knockdown of miR-551b-5p significantly increased CD2AP expression, and CD2AP knockdown effectively restored its expression level. **B **and** C** Co-transfected miR-551b-5p inhibitor and si-CD2AP significantly suppressed MPC-5 cells’ proliferation and induced apoptosis again. **D** CD2AP knockdown significantly upregulated the levels of inflammatory mediators IL-6 and TNF-α **E** Knockdown of CD2AP increased MDA levels and suppressed SOD levels. (^&&&^*P* < 0.001 VS control; ^###^*P* < 0.001 vs. inhibitor-NC; ***P < 0.001, ****P < 0.0001 vs. miR-551b-5p inhibitor + si-NC)

The expression of CD2AP was potently reduced upon LPS stimulation. However, knockdown of miR-551b-5p significantly increased CD2AP expression, and CD2AP knockdown effectively restored its expression level (Fig. 4A). Co-transfected miR-551b-5p inhibitor and si-CD2AP significantly suppressed MPC-5 cells proliferation and induced apoptosis again (Fig. 4B, C). CD2AP knockdown significantly upregulated the levels of inflammatory mediators IL-6 and TNF-α (Fig. 4D). Knockdown of CD2AP significantly increased MDA levels and suppressed SOD levels (Fig. 4E).

## Discussion

PNS severely impedes normal growth and development in pediatric patients. Without timely treatment, it may lead to complications such as infections, hypercoagulable states, and tubular injury. Severe cases may progress to renal failure, ultimately resulting in death [14]. miRNAs are now widely recognized not only as disease markers but also as playing a crucial role in treatment response. miR-551b-5p has been demonstrated to influence the onset and progression of various diseases. For example, Wei et al. demonstrated that miR-551b-5p may regulate autophagy impairment bidirectionally via the IL-6/STAT3 signaling pathway, thereby regulating the inflammatory response in acute pancreatitis [15]. Jin et al. found that miR-551b-5p is downregulated in the diabetic heart and its overexpression exhibits anti-fibrotic [16]. Our research indicates that miR-551b-5p expression is upregulated in PNS and the poor prognosis subgroup, suggesting that high miR-551b-5p expression is linked to the development and poor prognosis of PNS.

Although renal biopsy can assess renal prognosis, it is not routinely recommended for children with PNS due to the risk of complications [17]. Therefore, exploring new prognostic targets is crucial for the early identification and personalized treatment of high-risk PNS children. Research has shown that differential expression of miRNAs can distinguish steroid-sensitive from steroid-resistant NS [18]. And Wu et al. demonstrated that reduced serum CTLA-4 and increased S100A12 levels are correlated with malignant progression and poor prognosis of pediatric PNS [19]. In the study, miR-551b-5p effectively distinguished PNS patients from healthy controls. Furthermore, miR-551b-5p is an independent risk factor for predicting poor prognosis in PNS by multivariate logistic regression. These findings indicate that miR-551b-5p is a promising biomarker for assessing the severity of PNS and predicting poor prognosis. However, miR-551b-5p exhibits low specificity in predicting poor prognosis, at merely 56.58%, suggesting a relatively high false-positive rate. This indicates limitations in using miR-551b-5p as an independent prognostic indicator for poor outcomes in PNS. Future research will explore the combined predictive potential of miR-551b-5p alongside its downstream target genes and clinical indicators, thereby enhancing predictive accuracy.

miR-551b-5p expression positively correlated with TC, TG, BUN, SCr, and 24-h urinary protein, while it was negatively correlated with Alb. These factors are key clinical indicators for assessing the onset and prognosis of PNS. Hyperlipidemia, characterized by elevated TC and TG levels, is one of the clinical manifestations of NS [20]. Low serum Alb, elevated 24-h urinary protein, or increased SCr have been identified as risk factors in children with PNS, while BUN correlates with acute kidney injury [21, 22]. Furthermore, the Alb clearance estimated from Alb and 24-h urinary protein excretion predicts the risk of recurrence in minimal change disease [23]. miR-551b-5p expression showed significant correlations with these indicators, suggesting that its abnormally high expression is associated with lipid metabolism abnormalities, serum protein expression disorders, and renal impairment in patients with PNS. It may contribute to the progression of PNS by regulating serum or lipid levels and affecting renal function.

The underlying pathophysiology of PNS remains incompletely understood. Research has shown that pediatric PNS is linked to immune dysregulation and aberrant autoimmune activity. Perturbations in immune homeostasis may trigger persistent and excessive inflammation, ultimately resulting in structural and functional damage to renal tissues [24, 25]. As well-known pro-inflammatory factors, the levels of IL-6 and TNF-α increase with the severity of inflammation and infection, and they trigger substantial proliferative expansion in human regulatory T cells, without impairing their lineage stability or immunosuppressive function [26]. MDA serves as a biomarker for the degree of damage caused by oxidative stress in cells. SOD can eliminate free radicals and protect cells from oxidative damage [27]. Therefore, in response to oxidative stress, cells exhibit a rise in MDA alongside a reduction in SOD activity. We established an in vitro cellular model by inducing MPC-5 cells with LPS. The results indicate that miR-551b-5p knockdown promotes podocyte proliferation and reduces apoptosis, improves intracellular inflammatory responses and oxidative damage, thereby protecting renal function. It is speculated that elevated miR-551b-5p expression may exacerbate renal tissue structural destruction and injury in pediatric PNS patients by inducing renal inflammatory and oxidative stress responses, thereby leading to the development of PNS and poor prognosis.

Furthermore, the potential mechanism by which miR-551b-5p promotes PNS progression was explored. Research indicates that miRNA targeting mRNA influences the progression of diabetic nephropathy by regulating inflammation, fibrosis, oxidative stress, and apoptosis [28]. This study confirms that miR-551b-5p directly targets and regulates CD2AP. Numerous studies indicate that miRNA and CD2AP jointly influence the onset and progression of renal disease. For example, Ming et al. revealed that miR-182-5p induces excessive podocyte apoptosis and promotes diabetic nephropathy progression by targeting CD2AP [29]. Wang et al. reported that miR-939-5p contributes to the pathogenesis of nephrotic syndrome by suppressing the recruitment of RNA polymerase II to the CD2AP gene promoter [30]. Our results indicate that silencing CD2AP effectively reverses the effects of miR-551b-5p knockdown in promoting podocyte proliferation and inhibiting apoptosis, and exacerbates inflammation and oxidative stress-mediated podocyte injury. This study reveals a novel mechanistic insight into PNS pathogenesis, demonstrating that the co-regulation of podocyte function and inflammatory injury by miR-551b-5p and CD2AP could inform future diagnostic and prognostic strategies.

This study employed LPS-induced MPC-5 cells to establish an in vitro model of sepsis-associated renal inflammatory response, but it has certain limitations: due to the complexity of the PNS condition, renal injury may result from multiple factors beyond sepsis, including infection, allergic reactions, and nephrotoxic substances. However, the LPS model reflects only inflammation-mediated renal injury mechanisms and cannot comprehensively cover the multifaceted pathophysiological processes of PNS. The stability of the interaction mechanism between miR-551b-5p and CD2AP in PNS patients will be further validated through in vitro experiments (animal studies) to further validate, and their roles in different disease stages will also be examined. Moreover, several cutting-edge RNA technologies that have emerged in recent years point the way forward for future research. For instance, bio-nanopore technology has significantly enhanced the efficiency and accuracy of biomolecular detection [31]. Functional RNA structures are central to gene regulation and represent novel pathways for exploring disease pathogenesis [32]. And RNA base editing technology holds promise for developing precision therapies targeting distinct pathological subtypes [33]. Overall, the key to future research lies in applying advanced RNA technologies to translate miRNA discoveries into innovative diagnostic tools, ultimately enabling precision medicine for PNS.

## Conclusion

In conclusion, miR-551b-5p is significantly upregulated while CD2AP is significantly downregulated in PNS. miR-551b-5p serves as a biomarker for the pathogenesis and poor prognosis of pediatric PNS. Elevated miR-551b-5p expression inhibits podocyte proliferation and induces apoptosis, promotes renal inflammation and oxidative stress, thereby exacerbating renal tissue damage and driving the malignant progression of PNS, with its underlying mechanism potentially involving targeting CD2AP.

## Supplementary Information

Supplementary Material 1.

Supplementary Material 2.

## Data Availability

All data generated or analyzed during this study are included in this article. Further enquiries can be directed to the corresponding author.
