# Supplementary material for: Clinical value of miR-551b-5p in children with primary nephrotic syndrome and its regulatory role in disease progression
Source: Hereditas. 2026 Feb 9;163:41. doi: 10.1186/s41065-026-00644-3 (PMC12983489; doi:10.1186/s41065-026-00644-3)
Supplement: Supplementary file 2 — Supplementary Material 2. [file 41065_2026_644_MOESM2_ESM.pdf]

revised manuscript with track changes.查重.docx

作者为 znuura fonuzn

提交日期: 2025年12月04日 03:38下午 (UTC+0800)
提交作业代码: 2834205538
文档名称: revised_manuscript_with_track_changes.查重.docx (80.97K)
文字总数: 3811
字符总数: 23807

Clinical value of miR-551b-5p in children with primary nephrotic syndrome and its regulatory role in disease progression

Running title: The role of miR-551b-5p in pediatric PNS

Abstract

Objective: This research aims to explore the role of miR-551b-5p in the occurrence and progression of primary nephrotic syndrome (PNS) and its underlying mechanisms.

Methods: The study was conducted on a total of 107 PNS patients and 99 healthy volunteers (HV). And the PNS group was further divided into subgroups with favorable (n=76) and poor (n=31) prognosis. The expression of miR-551b-5p in the PNS and the poor prognosis group was quantified using qRT-PCR. The predictive capability of miR-551b-5p for the occurrence and poor prognosis of PNS was evaluated. The effects of miR-551b-5p knockdown on podocyte growth and inflammatory injury were examined. The interaction between miR-551b-5p and CD2AP was verified via database and luciferase assay.

Results: miR-551b-5p is obviously elevated in PNS and the poor prognosis group. miR-551b-5p exhibits strong diagnostic capability for both the onset and poor prognosis of pediatric PNS. High miR-551b-5p expression is an independent risk factor for poor prognosis in pediatric PNS patients. miR-551b-5p downregulation potently promotes podocyte proliferation and reduces apoptosis, and improves intracellular inflammatory responses and oxidative stress levels. Furthermore, CD2AP is a direct target of miR-551b-5p, and this regulatory axis synergistically contributes to the pathogenesis and progression of PNS.

Conclusion: miR-551b-5p is a potential biomarker for predicting the occurrence and poor prognosis of PNS. miR-551b-5p promotes the onset and progression of PNS by targeting CD2AP.

Keywords: miR-551b-5p; CD2AP; Primary nephrotic syndrome; Biomarker; Podocyte

1. Introduction

Primary nephrotic syndrome (PNS) is a highly prevalent glomerular disorder. It is responsible for about 90% of all pediatric nephrotic syndrome (NS) cases[1]. It is characterized by massive proteinuria, hypoalbuminemia, edema, and hyperlipidemia resulting from increased permeability of the glomerular filtration membrane to plasma albumin (Alb)[2, 3]. Glucocorticoids are the primary treatment for PNS. Despite a generally favorable response in most children, a significant proportion experience complications such as recurrent relapses, glucocorticoid dependence, or even resistance. And these patients are also at high risk of developing end-stage renal disease[4]. Therefore, identifying biomarkers associated with PNS is crucial for early diagnosis and prognostic management.

MicroRNAs (miRNAs) as biomarkers have shown great potential in the pathogenesis and prognosis of NS. For instance, Wang et al. reported that elevated urinary exosomal miR-193a correlates with increased incidence of primary focal segmental glomerulosclerosis in childhood NS and heightened risk of poor prognosis[5]. Xu et al. indicated that miR-151-3p influences the onset and progression of neurological disorders by targeting GLCCI1[6]. And Feng et al. show that miR-23b-3p and miR-30a-5p in urinary exosomes are promising biomarkers for the diagnosis and prognostic monitoring of PNS[7]. Research indicates that the upregulation of miR-551b-5p may be linked to poorer prognosis in childhood NS[8]. However, its specific role and mechanism have not been fully proven. Therefore, studying miRNAs and their molecular mechanisms may help provide new insights for predicting the occurrence and adverse outcomes of PNS.

CD2-associated protein (CD2AP), encoded by the CD2AP gene, is a key component of the glomerular filtration barrier and plays a crucial role in the structure and function of podocytes[9]. Dysregulation of CD2AP expression is associated with the pathogenesis of multiple diseases. For instance, Li et al.

reported that CD2AP contributes to the formation of a tumor microenvironment with weakened stroma in gastric cancer, thereby enhancing the efficacy of immunotherapy[10]. Yan et al. revealed that CD2AP deficiency leads to p38 MAPK activation, which subsequently aggravates the phenotypic and pathological manifestations of Alzheimer's disease[11]. According to Zhang et al., CD2AP facilitates the progression of glioblastoma multiforme by activating TRIM5-dependent NF-kB signaling[12]. Research has linked CD2AP gene mutations to the development of focal segmental glomerulosclerosis and identified them as a potential prognostic biomarker in renal clear cell carcinoma[13, 14]. Database analyses revealed that CD2AP is a target gene of miR-551b-5p. It was hypothesized that miR-551b-5p and CD2AP jointly influence podocyte function and inflammatory injury, which was discussed in the study results.

In this study, the clinical value of serum miR-551b-5p in predicting the onset and prognosis of PNS has been explored. The influence of miR-551b-5p on podocyte proliferation, apoptotic activity, and inflammatory injury via cell experiments, aiming to elucidate its contribution to the pathogenesis of PNS. Furthermore, the regulatory relationship between miR-551b-5p and its downstream target CD2AP was confirmed using bioinformatics prediction and dual-luciferase reporter assays. Their collaborative role in the development of PNS was further explored to provide novel targets for PNS diagnosis and prognostic evaluation.

2. Materials and methods

2.1 Clinical samples

Peripheral venous blood was collected from 107 PNS patients and 99 healthy volunteers (HV) at Shanxi Children’s Hospital from April 2019 to May 2021. Blood samples were collected from pediatric patients with PNS on the morning of the day following admission (after diagnosis and before initiating any immunosuppressive therapy). This study protocol was reviewed and approved by [the Ethics Committee

of Shanxi Children's Hospital and Shanxi Children's Hospital], approval number [No. ChiCTR2200016583]. Informed consent was obtained in writing from the legal guardian of each participant. The inclusion criteria comprised: (a) The patient presented with massive proteinuria, hypoalbuminemia, edema, and hyperlipidemia. And renal biopsy findings met the diagnostic criteria for PNS[15]; (b) The patient does not have secondary nephrotic syndrome or any other type of nephropathy. (c) The patient was newly diagnosed with PNS, had not taken any medications such as immunosuppressants, lipid-lowering agents, or hormones before the onset of symptoms. (d) Age ≤12 years old. (e) Undergoing glucocorticoid therapy, with no contraindications to treatment (recurrent relapses, glucocorticoid dependence, or even resistance). And exclusion criteria: (a) Familial dyslipidemia; (b) Patients with concomitant autoimmune diseases or malignancies; (c) Patients with concomitant cardiovascular or cerebrovascular diseases, or severe infections; (d) Patients with impaired cardiac, hepatic, or pulmonary function.

2.2 Follow-up and prognostic assessment

The prognosis of PNS children was followed up for 3 years after treatment by telephone or outpatient review. Based on follow-up criteria and PNS outcome standards, 107 PNS patients were categorized into a favorable prognosis subgroup (partial remission, complete remission, and clinical cure) and an unfavorable prognosis subgroup (no remission). The basis for prognosis assessment is as follows[15]: (1) Partial remission. Morning urine protein reduction >50%, serum Alb>25 g/L, renal function essentially stable. (2) Complete remission. Normal blood biochemistry and urinalysis. (3) Clinical cure. Long-term stable renal function with no recurrence for over 3 years after discontinuing medication. (4) No remission. Morning urine protein reduction <50%, persistent deterioration of renal function.

2.3 Cell culture and transfection

The MPC-5 cell suspension was sourced from EK Bioscience (Shanghai, China, Cat# CC-Y1716) and cultured in DMEM medium (Gibco, Cat# 11966025) supplemented with 10% FBS (Gibco, Cat# 10091148), 100 U/mL penicillin, and 100 μg/mL streptomycin at 37°C under 5% CO_2. After 7 days, inflammatory injury was induced by treating the cells with 1 μg/mL lipopolysaccharide (LPS; Sigma-Aldrich, USA, Cat# L2630) for 12 h. miR-551b-5p mimics (GAAAUCAAGCGUGGGUGAGACC), miR-551b-5p inhibitors (GGUCUCACCCACGCUUGAUUUC), CD2AP siRNA, and corresponding negative controls (NC, 5'-CAGUACUUUUGUGUAGUACAA-3') were obtained from RiboBio (Guangzhou, China) and transfected into podocytes using Lipofectamine 2000 (Invitrogen, Thermo Fisher Scientific, Cat# 11668019).

2.4 Extraction of total RNA and qRT-PCR

After allowing the blood to stand at room temperature for 30 min, it was centrifuged at 3000 rpm for 10 min at 4°C using a refrigerated ultracentrifuge (Beckman Coulter Optima XPN-100 ultracentrifuge, Beckman Coulter). The resulting serum was then aliquoted and stored at -80°C for subsequent use. All samples were thawed only once. RNA quality was assessed using the Agilent 2100 Bioanalyzer with the RNA Nano 6000 chip, confirming normal profiles. TRIzol reagent (Solarbio, Beijing, China, Cat# R1100) was applied for extracting total RNA. Free hemoglobin absorbance in samples was measured at 414 nm using a NanoDrop 1000 spectrophotometer (Thermo Scientific, Cat# ND2000). A threshold of >0.2 arbitrary units at A414 was set to indicate hemolysis. All hemolyzed samples were excluded from final analysis to ensure miRNA profiles remained unaffected by erythrocyte-derived miRNAs. cDNA was synthesized and qPCR detection for miR-551b-5p was conducted via the TaqMan reverse transcription kit (ThermoFisher Scientific, Cat# 4366596) and TaqMan miRNA Assays (Thermo Fisher Scientific, Cat# 4427975). Reverse transcription and the mRNA level of CD2AP using M-MLV Reverse

Transcriptase (Thermo Fisher Scientific, Invitrogen, Cat# 28025013) and 2×SYBR Green PCR Mastermix (Solarbio, Beijing, China, Cat# SY102). The reverse transcription conditions were 25°C for 30 min, 42°C for 30 min, and subsequently 85°C for 5 min. And the PCR amplification was performed using the following conditions: 95°C for 30s, followed by 39 cycles at 95°C for 5s, 60°C for 30s, and 72°C for 15s. miR-551b-5p (forward primer 5'-GTCGTATCCAGTGCAGGGTCCGAGGTATTCGCACTGGATACGACGGTCTC-3' and reverse primer 5'-GCAGGGTCCGAGGTATTC-3'); CD2AP (forward primer 5'-CAAGATGCCTGGAAGACGA-3' and reverse primer 5'-GCACTTGAAGGTGTTGAAAGAG-3'). Next, U6 (forward 5′-CTCGCTTCGGCAGCACA-3′ and reverse 5′-AACGCTTCACGAATTTGCGT-3′)and GAPDH (forward 5'-ACTGGCATGGCCTTCCGT-3' and reverse 5'-CCACCCTGTTGCTGTAGCC-3') were used as controls for miR-551b-5p and CD2AP. All amplifications in this study exhibited efficiency ranging from 90% to 110%, with the linear regression correlation coefficient of the standard curve exceeding 0.990. And their expressions were quantified through the $2^{-\Delta\Delta ct}$ method. All experiments were independently replicated at least three times to ensure reproducibility of results. The intra-batch CV averaged 0.8%. The inter-batch CV for quality control samples was 7.2%.

2.5 Dual-luciferase reporter assay

The wild-type (WT) or mutated (MUT) sequences of miR-551b-5p containing the binding sites of CD2AP were designed by Gene Pharma (China) and inserted into the pGL3 luciferase vector (Promega, Madison, WI, USA, Cat# E1751). MPC-5 cells were co-transfected with miR-551b-5p-WT/MUT and CD2AP inhibitor/ CD2AP mimic/mimic-NC/inhibitor-NC by lipofectamine 2000 (Invitrogen, Thermo Fisher Scientific, Cat# 11668019). Cell lysates were collected after 48 h of transfection, and the relative

luciferase activity was quantified by normalizing firefly luminescence to Renilla luminescence with the dual-luciferase reporting kit (Promega, Shanghai, China, Cat# E1910). Each independent experiment included three biological replicates.

2.6 CCK8 assay

The transfected cells (1×10^4) were trypsinized, seeded into 96-well plates, and incubated for 24, 48, or 72 h. Subsequently, 10 µL of CCK-8 reagent was added to each well, followed by incubation at 37 °C for 2 h. Cell viability was measured using a CCK-8 assay kit (Beyotime, Shanghai, China, Cat# C0042), and the absorbance at 450 nm was detected with a microplate reader (BMG LABTECH, Offenburg, Germany).

2.7 Flow cytometry

The Annexin V-FITC Apoptosis Detection Kit (BD Biosciences) was used to evaluate apoptosis in MPC-5 cells. After 48 h of transfection, the cells were trypsinized, harvested, and stained with Annexin V-FITC and propidium iodide (PI) for 15 min under light-protected conditions. Subsequently, the cells were centrifuged, washed twice with PBS, and analyzed by FACScan flow cytometry (BD Biosciences, USA) within 1 h.

2.8 Detection of inflammation and oxidative stress index

LPS-stimulated MPC-5 cells were centrifuged at approximately 10^6 cells per tube, resuspended in 450 µL PBS, homogenized, and then centrifuged at 10,000 rpm for 10 min at 4°C. The collected supernatant was stored on ice. The concentrations of interleukin-6 (IL-6) and tumour necrosis factor-α (TNF-α) were quantified using enzyme-linked immunosorbent assay (ELISA) kits from the Nanjing Institute of Bioengineering, China. Meanwhile, the levels of malondialdehyde (MDA) and the activity of superoxide dismutase (SOD) were measured with ELISA kits supplied by Wuhan Saipei Biotechnology Co., Ltd.,

China. Following cessation of the color development, a microplate reader (BMG LABTECH, Offenburg, Germany) was employed to assess the optical density at 450 nm. All measurements were performed in triplicate for each well.

2.9 Statistical Analysis

All data were processed using SPSS version 23 (New York, USA) and GraphPad Prism 10.0 (California, USA). Intergroup clinical and pathological characteristics were analyzed using chi-square and t-tests. The association between miR-551b-5p and CD2AP expression was assessed using Pearson's correlation coefficient. ROC analysis revealed the diagnostic value of miR-551b-5p, and multivariate logistic regression assessed its predictive value for poor prognosis in PNS. The analysis of two-group comparisons relied on t-tests. Univariate ANOVA was employed for multiple comparisons, while two-way ANOVA compared cell proliferation capacity. Bonferroni's correction for multiple comparisons.

3. Results

3.1 The relative expression of miR-551b-5p and its diagnostic value

A pronounced elevation in miR-551b-5p was discovered in PNS compared to HV (Fig.1A). The 107 children with PNS were further divided into groups with favorable and poor prognoses. Compared with the group with favorable prognosis, the expression of miR-551b-5p was markedly upregulated in the group with poor prognosis (Fig.1B). Serum miR-551b-5p can distinguish PNS patients from healthy individuals with high accuracy (AUC=0.787, 95% CI: 0.725-0.848), the sensitivity and specificity were 82.24% and 66.67%, respectively (Fig.1C). Moreover, serum miR-551b-5p also showed significant predictive value for adverse outcomes in pediatric PNS patients (AUC=0.734, 95% CI: 0.634-0.833), sensitivity was 83.87%, specificity was 56.58% (Fig.1D).

3.2 Comparison of clinical characteristics between HV and patients with PNS

Clinical data for HV and PNS groups are presented in Table 1. There were no significant differences between the HV and the PNS group in terms of gender, age, and BMI ($P > 0.05$). However, compared with the HV, the PNS group exhibited higher levels of total cholesterol (TC), triglycerides (TG), blood urea nitrogen (BUN), serum creatinine (SCr), and 24-h urinary protein, as well as lower levels of Alb.

3.3 Correlation of serum miR-551b-5p expression with serum, blood lipids, and renal function markers

Pearson correlation analysis revealed that serum miR-551b-5p levels were positively correlated with blood lipid markers (TC and TG) and renal function markers (BUN, SCr, or 24-h urinary protein), and significantly negatively correlated with serum Alb levels ($P < 0.001$, Table 2).

3.4 Comparison of clinical characteristics in children with PNS of different prognoses

Among 107 children with PNS, 31 cases showed no remission; 26 achieved partial remission, 29 attained complete remission, and 21 were clinical cures. Consequently, the 107 cases were categorized into a favorable prognosis group (76 cases) and a poor prognosis group (31 cases). As shown in Table 3, in the poor prognosis subgroup, BUN and 24-h urine protein were all higher than in the favorable prognosis subgroup, while serum Alb levels were lower.

3.5 Factors influencing poor prognosis in children with PNS

Multivariate logistic regression analysis was performed using the prognosis of children with PNS as the dependent variable (favorable = 0, poor = 1), with variables showing significant differences in Table 3 as independent variables. Results indicated that elevated miR-551b-5p levels were independent risk factors for poor prognosis in pediatric PNS patients (Table 4).

3.6 Effects of miR-551b-5p knockdown on Podocyte growth and inflammatory injury

LPS stimulated MPC-5 cells to simulate an inflammatory injury model. miR-551b-5p expression is significantly upregulated in LPS-induced MPC-5 cells, while miR-551b-5p knockdown partially

reversed the effect (Fig 2A). The results of the CCK-8 assay and flow cytometry showed that LPS stimulation significantly inhibited MPC-5 cells' proliferation and induced apoptosis, while knockdown of miR-551b-5p significantly restored cell proliferation and reduced apoptosis (Fig. 2B, C). Additionally, ELISA results revealed that the relative expression of inflammatory mediators (IL-6 and TNF-α) significantly increased under LPS induction, whereas miR-551b-5p knockdown significantly restored their levels (Fig. 2D, E). Following LPS stimulation, MDA levels significantly increased while SOD activity markedly decreased. However, miR-551b-5p knockdown significantly restored the expression of these oxidative stress markers (Fig. 2F, G).

3.7 Interaction of miR-551b-5p with CD2AP

miR-551b-5p could directly bind to the 3′UTR of CD2AP via the TargetScan database (https://www.targetscan.org/vert_72/) (Fig 3A). miR-551b-5p expression is negatively related to CD2AP expression (r=−0.720, P<0.001, Fig 3B). CD2AP expression was substantially reduced in PNS compared to HV (Fig. 3C). The miR-551b-5p mimic markedly suppresses CD2AP luciferase activity, while the miR-551b-5p inhibitor leads to a significant increase. However, following a mutation in CD2AP, the luciferase activity was not affected (Fig. 3D).

3.8 miR-551b-5p and CD2AP jointly influence podocyte function and inflammatory injury

The expression of CD2AP was potently reduced upon LPS stimulation. However, knockdown of miR-551b-5p significantly increased CD2AP expression, and CD2AP knockdown effectively restored its expression level (Fig. 4A). Co-transfected si-miR-551b-5p and CD2AP inhibitor significantly suppressed MPC-5 cells proliferation and induced apoptosis again (Fig. 4B, C). CD2AP knockdown significantly upregulated the levels of inflammatory mediators IL-6 and TNF-α (Fig. 4D). Knockdown of CD2AP significantly increased MDA levels and suppressed SOD levels (Fig. 4E).

4. Discussion

PNS severely impedes normal growth and development in pediatric patients. Without timely treatment, it may lead to complications such as infections, hypercoagulable states, and tubular injury. Severe cases may progress to renal failure, ultimately resulting in death[16]. miRNAs are now widely recognized not only as disease markers but also as playing a crucial role in treatment response. miR-551b-5p has been demonstrated to influence the onset and progression of various diseases. For example, Wei et al. demonstrated that miR-551b-5p may regulate autophagy impairment bidirectionally via the IL-6/STAT3 signaling pathway, thereby regulating the inflammatory response in acute pancreatitis[17]. Dong et al. indicated that dysregulation of miR-551b-5p is associated with an unfavorable prognosis in thyroid cancer and enhances tumor cell migration and invasion[18]. Jin et al. found that miR-551b-5p is downregulated in diabetic cardiac tissue and that its overexpression attenuates fibrosis[19]. Our research indicates that miR-551b-5p expression is significantly upregulated in patients with PNS, and it exhibited a marked increase in the poor prognosis subgroup compared to the favorable prognosis subgroup. This indicates that the abnormal elevation of miR-551b-5p expression is linked to the development and poor prognosis of PNS.

Although renal biopsy can assess renal prognosis, it carries potential risks of complications and is generally not recommended for children with PNS[20]. Therefore, exploring new prognostic targets is crucial for the early identification and personalized treatment of high-risk PNS children. Research has shown that miRNAs differentially regulate steroid-responsive and steroid-resistant NS, serving as biomarkers for detecting these distinct disease categories and participating in the investigation of pathological mechanism pathways[21]. And Wu et al. demonstrated that reduced serum CTLA-4 and increased S100A12 levels in pediatric PNS are correlated with advancing disease and poor outcomes[22].

In the study, miR-551b-5p effectively distinguished PNS patients from healthy controls. And it performs well in predicting poor prognosis. Furthermore, multivariate logistic regression analysis indicated that other clinical indicators showed no significant differences in predicting poor PNS prognosis, but miR-551b-5p emerged as an independent risk factor. These findings indicate 7 that miR-551b-5p holds promise as a biomarker for assessing the severity of PNS and predicting adverse outcomes.

Pearson correlation analysis revealed that miR-551b-5p positively correlated with TC, TG, BUN, SCr, and 24-hour urinary protein in patients with PNS, while it was negatively correlated with Alb. These factors are associated with adverse outcomes in kidney disease. Hyperlipidemia, characterized by elevated TC and TG levels, is one of the clinical manifestations of NS[23]. Low serum Alb, elevated 24-h urinary protein, or increased SCr have been identified as risk factors in children with PNS, while BUN and 24-h urinary protein levels have also been linked to 8 acute kidney injury in patients diagnosed with nephropathy[24, 25]. Furthermore, the Alb clearance estimated from Alb 8 and 24-h urinary protein excretion predicts the risk of recurrence in minimal change disease[26]. miR-551b-5p expression showed significant correlations with these indicators, suggesting that its abnormally high expression is associated with lipid metabolism abnormalities, serum protein expression disorders, and renal impairment in patients with PNS. It may contribute to the progression of PNS by regulating serum or lipid levels and affecting renal function.

The underlying pathophysiology of PNS remains incompletely understood. Research has shown that pediatric PNS is linked to immune dysregulation and aberrant autoimmune activity. Perturbations in immune homeostasis may trigger persistent and excessive inflammation, ultimately resulting in structural and functional damage to renal tissues[27, 28]. As well-known pro-inflammatory factors, the levels of IL-6 and TNF-α increase with the severity of inflammation and infection, and they trigger substantial

proliferative expansion in human regulatory T cells, without impairing their lineage stability or immunosuppressive function[29]. MDA serves as a biomarker for the degree of damage caused by oxidative stress in cells. SOD can eliminate free radicals and protect cells from oxidative damage[30]. Therefore, in response to oxidative stress, cells exhibit a rise in MDA alongside a reduction in SOD activity. We established an in vitro cellular model by inducing MPC-5 cells with LPS. The results indicate that miR-551b-5p knockdown promotes podocyte proliferation and reduces apoptosis, improves intracellular inflammatory responses and oxidative damage, thereby protecting renal function. It is speculated that elevated miR-551b-5p expression may exacerbate renal tissue structural destruction and injury in pediatric PNS patients by inducing renal inflammatory and oxidative stress responses, thereby leading to the development of PNS and poor prognosis.

Furthermore, the possible mechanism by which miR-551b-5p promotes PNS progression was explored. Research indicates that dysregulated miRNAs modulate various cellular pathways by targeting mRNAs, including inflammation, fibrosis, oxidative stress, and apoptosisthere by altering the progression of diabetic nephropathy[31]. This study confirms that miR-551b-5p directly targets and regulates CD2AP. Research has demonstrated that miRNA and CD2AP jointly influence the onset and progression of renal disease. For example, Ming et al. revealed that miR-182-5p induces excessive podocyte apoptosis and promotes diabetic nephropathy progression by targeting CD2AP[32]. Wang et al. reported that miR-939-5p contributes to the pathogenesis of nephrotic syndrome by suppressing the recruitment of RNA polymerase II to the CD2AP gene promoter[33]. Our results indicate that silencing CD2AP effectively reverses the effects of miR-551b-5p knockdown in promoting podocyte proliferation and inhibiting apoptosis, and exacerbates inflammation and oxidative stress-mediated podocyte injury. This study reveals a novel mechanistic insight into PNS pathogenesis, demonstrating that the co-regulation of

podocyte function and inflammatory injury by miR-551b-5p and CD2AP could inform future diagnostic and prognostic strategies.

This study employed LPS-induced MPC-5 cells to establish an in vitro model of sepsis-associated renal inflammatory response, but has certain limitations: due to the complexity of the PNS condition, renal injury may result from multiple factors beyond sepsis, including infection, allergic reactions, and nephrotoxic substances. However, the LPS model reflects only inflammation-mediated renal injury mechanisms and cannot comprehensively cover the multifaceted pathophysiological processes of PNS. The stability of the interaction mechanism between miR-551b-5p and CD2AP in PNS patients will be further validated through in vitro experiments, such as animal studies, to further validate, and their roles in different disease stages will also be examined. Moreover, several cutting-edge RNA technologies that have emerged in recent years point the way forward for future research. For instance, bio-nanopore technology has significantly enhanced the efficiency and accuracy of biomolecular detection[34]. Functional RNA structures are central to gene regulation and represent novel pathways for exploring disease pathogenesis[35]. And RNA base editing technology holds promise for developing precision therapies targeting distinct pathological subtypes[36]. Overall, future research should focus on the application of advanced RNA technologies to translate miRNA research findings into innovative diagnostic tools and therapeutic approaches, thereby advancing PNS diagnosis and treatment into the era of genuine precision medicine.

5. Conclusion

In conclusion, this study demonstrates that miR-551b-5p is significantly upregulated while CD2AP is significantly downregulated in patients with PNS. miR-551b-5p serves as a biomarker for the pathogenesis and poor prognosis of pediatric PNS. Elevated miR-551b-5p expression inhibits podocyte

proliferation and induces apoptosis, promotes renal inflammation and oxidative stress, exacerbating renal tissue damage. This ultimately leads to the malignant progression of PNS, with its underlying mechanism potentially involving targeting CD2AP.

revised manuscript with track changes.查重.docx

原创性报告

22%	17%	17%	4%
相似指数	网际网络来源	出版物	学生文稿

主要来源

1	www.frontiersin.org<br>网际网络来源	3%
2	Yong Xu, Yanli Wang. "lncRNA TMEM147-AS1 promotes acute myeloid leukemia development by regulating miR-873-3p/ZFX axis", Journal of Molecular Histology, 2025<br>出版物	2%
3	www.dovepress.com<br>网际网络来源	2%
4	www.spandidos-publications.com<br>网际网络来源	2%
5	www.ncbi.nlm.nih.gov<br>网际网络来源	1%
6	www.mdpi.com<br>网际网络来源	1%
7	Anbing Dong, Ming Gao, Xiangqian Zheng, Xianhui Ruan. "Dysregulation of miR-551b-5p and SETD2 Predicts Poor Prognosis and Promotes Migration and Invasion of Thyroid Cancers", Endocrine, Metabolic & Immune Disorders - Drug Targets, 2023<br>出版物	1%
8	"Poster Abstracts", American Journal of Transplantation, 2018<br>出版物	1%

9	Guoqiang Du, Xiaoqing Wang, Yidi Wu, Yongfei Zhang, Wei Liu, Rongde Wu. "Downregulation of miR-140-5p affects the pathogenesis of HSCR by targeting EGR2", Pediatric Surgery International, 2020<br>出版物	1%
10	Yongpeng Zhang, Liying Yan, Wang Han. "Elevated Level of miR-551b-5p is Associated With Inflammation and Disease Progression in Patients With Severe Acute Pancreatitis", Therapeutic Apheresis and Dialysis, 2018<br>出版物	1%
11	www.aging-us.com<br>网际网络来源	1%
12	Xiaomeng Jiang, Menglin Jiang, Min Xu, Jing Xu, Yi Li. "Identification of diagnostic utility and molecular mechanisms of circulating miR-551b-5p in gastric cancer", Pathology - Research and Practice, 2019<br>出版物	1%
13	tessera.spandidos-publications.com<br>网际网络来源	1%
14	molmed.biomedcentral.com<br>网际网络来源	<1%
15	Dan Li, Xuejiao Xie, Jie Wang, Yanjie Bian, Qingzhang Li, Xuejun Gao, Chunmei Wang. "MiR-486 Regulates Lactation and Targets the PTEN Gene in Cow Mammary Glands", PLOS ONE, 2015<br>出版物	<1%
16	Molecular Mechanisms in the Pathogenesis of Idiopathic Nephrotic Syndrome, 2016.<br>出版物	<1%

17	link.springer.com<br>网际网络来源	<1 %
18	www.science.gov<br>网际网络来源	<1 %
19	d.docksci.com<br>网际网络来源	<1 %
20	Linfu Li, Renbing Shi, Weimei Shi, Rui Zhang, Longhuo Wu. "Oxysophocarpine protects airway epithelial cells against inflammation and apoptosis by inhibiting miR-155 expression", Future Medicinal Chemistry, 2020<br>出版物	<1 %
21	Submitted to University of Glasgow<br>学生文稿	<1 %
22	pericles.pericles-prod.literatumonline.com<br>网际网络来源	<1 %
23	www.cell.com<br>网际网络来源	<1 %
24	www.omicsdi.org<br>网际网络来源	<1 %
25	www.degruyterbrill.com<br>网际网络来源	<1 %
26	www.researchgate.net<br>网际网络来源	<1 %
27	Ming Li, Zheng Zhi, Xuan Jiang, Guo-Cai Duan et al. "METTL9 derived circular RNA circ-METTL9 sponges miR-551b-5p to accelerate colorectal cancer progression by upregulating CDK6", Carcinogenesis, 2023<br>出版物	<1 %

28	Submitted to University of Queensland<br>学生文稿	<1 %
29	Submitted to University of Technology, Sydney<br>学生文稿	<1 %
30	cnjournal.biomedcentral.com<br>网际网络来源	<1 %
31	downloads.hindawi.com<br>网际网络来源	<1 %
32	rcastoragev2.blob.core.windows.net<br>网际网络来源	<1 %
33	Lotte Risom, Carsten Lundby, Jonas Juhl Thomsen, Lone Mikkelsen, Steffen Loft, Gitte Friis, Peter Møller. "Acute hypoxia and reoxygenation-induced DNA oxidation in human mononuclear blood cells", Mutation Research - Fundamental and Molecular Mechanisms of Mutagenesis, 2007<br>出版物	<1 %
34	academic.oup.com<br>网际网络来源	<1 %
35	actaneurocomms.biomedcentral.com<br>网际网络来源	<1 %
36	all-imm.com<br>网际网络来源	<1 %
37	assets-eu.researchsquare.com<br>网际网络来源	<1 %
38	tau.amegroups.com<br>网际网络来源	<1 %
39	"Abstracts", Journal of Gastroenterology and Hepatology, 2016	<1 %

出版物

不含引文	开	不含相符结果	关闭
排除参考书目	开		
